# Potential Role of Colchicine in Combating COVID-19 Cytokine Storm and Its Ability to Inhibit Protease Enzyme of SARS-CoV-2 as Conferred by Molecular Docking Analysis

**DOI:** 10.3390/medicina58010020

**Published:** 2021-12-23

**Authors:** Noha A. Kamel, Nasser S. M. Ismail, Ibrahim S. Yahia, Khaled M. Aboshanab

**Affiliations:** 1 Department of Microbiology, Faculty of Pharmacy, Misr International University (MIU), Cairo 19648, Egypt; noha.ahmed@miuegypt.edu.eg; 2 Department of Pharmaceutical Chemistry, Faculty of Pharmacy, Future University in Egypt (FUE), Cairo 11835, Egypt; nasser.saad@fue.edu.eg; 3 Laboratory of Nano-Smart Materials for Science and Technology (LNSMST), Department of Physics, Faculty of Science, King Khalid University, P.O. Box 9004, Abha 61413, Saudi Arabia; ihussein@kku.edu.sa; 4 Research Center for Advanced Materials Science (RCAMS), King Khalid University, P.O. Box 9004, Abha 61413, Saudi Arabia; 5 Nanoscience Laboratory for Environmental and Biomedical Applications (NLEBA), Semiconductor Laboratory, Department of Physics, Faculty of Education, Ain Shams University (ASU), Roxy, Cairo 11757, Egypt; 6 Department of Microbiology & Immunology, Faculty of Pharmacy, Ain Shams University (ASU), Abbassia, Cairo 11566, Egypt

**Keywords:** COVID-19, cytokine storm, colchicine, protease inhibitor, molecular docking, RNA polymerase, SARS-CoV-2

## Abstract

Despite the advance in the management of Coronavirus disease 2019 (COVID-19), the global pandemic is still ongoing with a massive health crisis. COVID-19 manifestations may range from mild symptoms to severe life threatening ones. The hallmark of the disease severity is related to the overproduction of pro-inflammatory cytokines manifested as a cytokine storm. Based on its anti-inflammatory activity through interfering with several pro and anti-inflammatory pathways, colchicine had been proposed to reduce the cytokine storm and subsequently improve clinical outcomes. Molecular docking analysis of colchicine against RNA-dependent RNA polymerase (RdRp) and protease enzymes of Severe Acute Respiratory Syndrome Coronavirus-2 (SARS-CoV-2) revealed that colchicine provided a grid-based molecular docking method, C-DOCKER interaction energy 64.26 and 47.53 (Kcal/mol) with protease and RdRp, respectively. This finding indicated higher binding stability for colchicine–protease complexes than the colchicine–RdRp complex with the involvement of seven hydrogen bonds, six hydrogen acceptors with Asn142, Gly143, Ser144, and Glu166 and one hydrogen-bond donors with Cys145 of the protease enzyme. This is in addition to three hydrophobic interactions with His172, Glu166, and Arg188. A good alignment with the reference compound, Boceprevir, indicated high probability of binding to the protease enzyme of SARS-CoV-2. In conclusion, colchicine can ameliorate the destructive effect of the COVID-19 cytokine storm with a strong evidence of antiviral activity by inhibiting the protease enzyme of SARS-CoV-2.

## 1. Introduction

Since its declaration as a pandemic on 11th of March 2020, Coronavirus disease 2019 (COVID-19) caused by Severe Acute Respiratory Syndrome Coronavirus-2 (SARS-CoV-2) had threatened the globe, leading to devastating consequences on public health. As of 13 August 2021, the World Health Organization (WHO) had reported over 205,338,159 million cases of infection with SARS-CoV-2 and over 4,333,094 million deaths [1]. Clinically, the progression of COVID-19 could be classified into mild, moderate, and severe cases ensuring its broad spectrum of clinical presentation that ranges from minimal symptoms where most patients completely recover to severe life-threatening viral pneumonia with respiratory failure leading to death [2]. The hallmark of COVID-19’s severity is mainly related to an exaggerated immune response, characterized by overproduction of pro-inflammatory cytokines and chemokines that may turn out to be a cytokine storm in some patients. Many of the innate immune system cells, such as macrophages, monocytes, dendritic cells, and neutrophils play a vital role in triggering signaling processes to express a variety of cytokines, namely interleukin (IL)-1, IL-6, IL-8, IL-10, tumor necrosis factor alfa (TNF-α), and interferon (IFN)-γ [3]. Accordingly, targeting such inflammatory pathways with broad anti-inflammatory agents may pave the way for ameliorating the destructive effect of the cytokine storm in severe COVID-19 cases.

Several millennia ago, colchicine, a naturally occurring tricyclic alkaloid extracted from the bulb of *Autumn colchicum,* had shown analgesic and anti-inflammatory properties, and was used to treat pain and swelling [4]. Over the years, colchicine had gained wide acceptance for the treatment of a variety of auto-inflammatory diseases including the prototypical inflammatory gout flares, familial Mediterranean fever, recurrent pericarditis, and many dermatologic diseases [4]. Based on current knowledge about colchicine interference with numerous inflammatory pathways, we hypothesize that its immunomodulatory effect can control hyper-inflammatory immune response in acute respiratory distress syndrome (ARDS) and extra-pulmonary multiple organ dysfunction (MOD) associated with COVD-19 cases.

## 2. Colchicine Mechanism of Action

**Figure 1:**
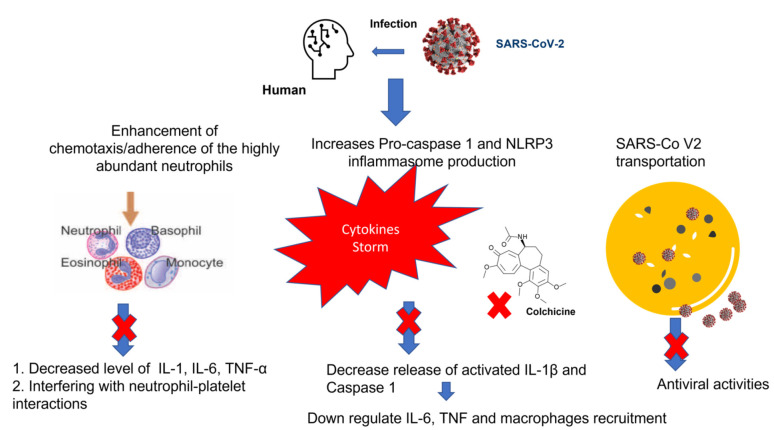
Mechanism of the anti-inflammatory activity of colchicine during infection with SARS-CoV-2. NLRP3 inflammasome (NOD-like receptor (NLR) subfamily pyrin domain containing 3 inflammasome); IL, Interleukin, TNF, Tumor necrosis factor.

Colchicine exhibits multifaceted mechanisms of action with anti-inflammatory and antiviral activity, showing great promise for alleviating ARDS and MOD manifested by cytokine storms in COVID-19 patients. Both anti-inflammatory and antiviral activity of colchicine depends on the disruption of the microtubule filaments [5]. As shown in Figure 1, the anti-inflammatory effect of colchicine is wide-ranging and primarily occurs through the interruption of tubulin, a protein that polymerizes into long chains forming microtubules that are responsible for cellular division, migration, signaling, and transport [6]. Additionally, colchicine can likely modulate the chemotaxis/adherence of the highly abundant neutrophils [7] and monocytes/macrophages to the lungs of COVID-19 patients [8]. Colchicine interferes with neutrophils, the primary cells involved in inflammation through inhibiting their migration to infected tissue, reducing their adherence to endothelium cells by suppressing the expression of E-selectin, an adhesion molecule, leading to decreased levels of IL-1, IL-6, and TNF-α [9,10], and reducing their production of superoxide free radicals [11]. On the level of reducing MOD in the circulatory system, colchicine can play a vital role in preventing atherothrombosis through interfering with neutrophil–platelet interactions, which is an influential step in the inflammatory reactions. Such interplay works on restricting platelets to inflammatory sites and provoke neutrophils to release neutrophil extracellular traps that are directly linked to thrombosis [12,13]. Moreover, a recent study reported on the selectivity of colchicine for hepatocytes and its ability to inhibit myeloid cell activation through the induction of hepatokines [14].

Additionally, colchicine act as powerful inhibitor of NLRP3 inflammasome (NOD-like receptor (NLR) subfamily pyrin domain containing 3 inflammasome), which is widely expressed on monocytes and macrophages. As a main component in innate immunity signaling, NLRP3 act as a pattern recognition receptor that is composed of a sensor, adaptor, and effector component which, upon inflammasome assembly/activation, leads to the release of activated IL-1β and IL-18 [15]. Notably, colchicine interference with IL-1β will consequently down regulate IL-6, TNF, and additional recruitment of macrophages [16].

The antiviral property of colchicine depends mainly on the destruction of the microtubular network that will subsequently interfere with the intracellular transportation of viral particles in host cells [17]. In addition to microtubules, other components of the cytoskeleton, such as actin and intermediate filaments, play a crucial role in viral pathogenesis. A recent study was conducted to explore the SARS-CoV-2 Spike glycoproteins for designing potential antiviral targets aiming to stem the COVID-19 pandemic [18]. Indeed, microtubule cytoskeleton is a chief player for coronaviruses’ spike glycoproteins to attach to cell surface [19], directing intracellular viral transport and assembly [20]. Additionally, studies have revealed that distress induced by viroporin E (an envelope protein with a tendency to assemble and create Ca^+2^ permeable ion channel) and another viroporin 3a can easily induce NLRP3 activation in the case of SARS-CoV-1 and 2 [21,22]. Hence, targeting the NLRP3 inflammatory pathway with colchicine can act as a prophylactic measure to slow down the progression of the cytokine storm.

Regarding the remarkably large SARS-CoV-2 RNA genome (30 Kb), it possesses the potential to encode several proteins that play crucial roles in viral replication. Upon coronavirus internalization and un-coating, its single-stranded positive RNA genome is released in the host cytoplasm and translated into the large viral polyprotein (pp) to mark the onset of a highly regulated complex process of viral gene expression [23]. The pp1a and pp1ab will further undergo autoproteolytic cleavage to give 10 and 16 nonstructural proteins (nsps), respectively [24]. Such cleavage is mediated by two viral proteases, main protease (Mpro) which is also known as 3-chyomotrypsin like protease (3CLpro) and papain like proteases (PLpro) to ensure pp processing into smaller components for folding and packaging in new virions. Out of 16 nsps, 11 are generated by the key enzyme Mpro that renders its inhibition an attractive target for developing anti-SARS-CoV drugs [25]. Of interest, there is no report yet in the literature on human protease analogue to viral Mpro, ensuring that targeting such viral protease will show minimal toxicity to host cell. In addition to this, most RNA viruses (except retroviruses) require the RNA dependent RNA polymerase (RdRp) for replication and transcription of their viral genome. Of concern, the RdRP active sites are the most accessible and conserved regions within RNA viruses [26]. Accordingly, targeting this enzyme may offer a broad range of effective antiviral agents. On the basis of its immunomodulatory activity and ability to interfere with the viral transportation to host cell, we (for the first time) have further explored the anti-SARS-Cov-2 activity of colchicine against RdRP and Mpro through computational structure-based drug discovery.

## 3. Molecular Docking Analysis of Colchicine against RNA-dependent RNA Polymerase and Protease Enzymes of SARS-CoV-2

**Figure 2:**
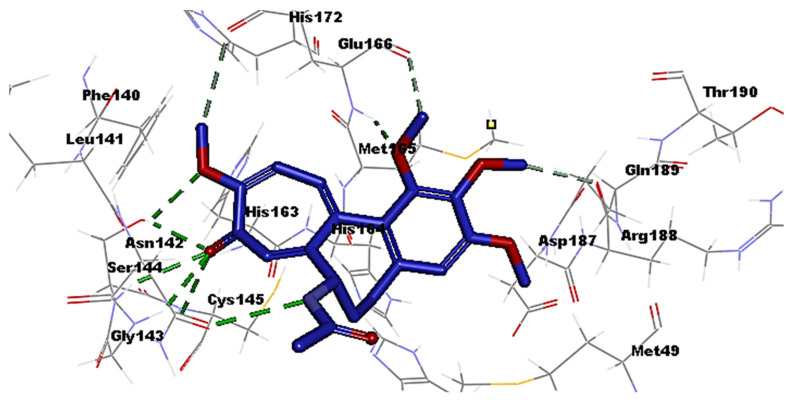
Interaction of Colchicine (blue) with the key amino acids of the protease enzyme of SARS-CoV-2, hydrogen bond (green) hydrophobic (grey).

**Figure 3:**
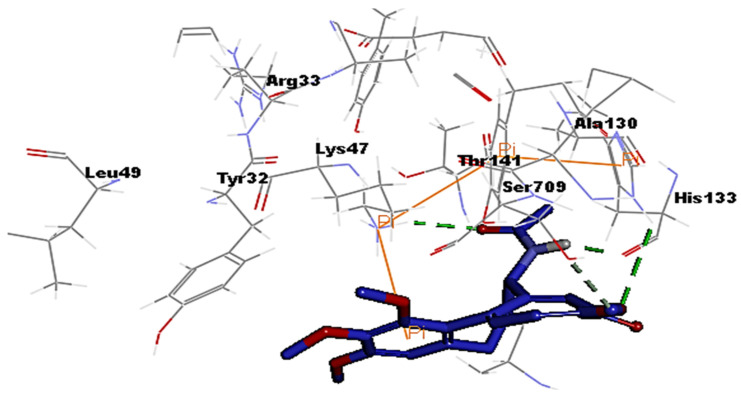
Interaction of Colchicine (blue) with the key amino acids of the polymerase (RdRp) enzyme of SARS-CoV-2, hydrogen bond (green) hydrophobic (orange).

**Figure 4:**
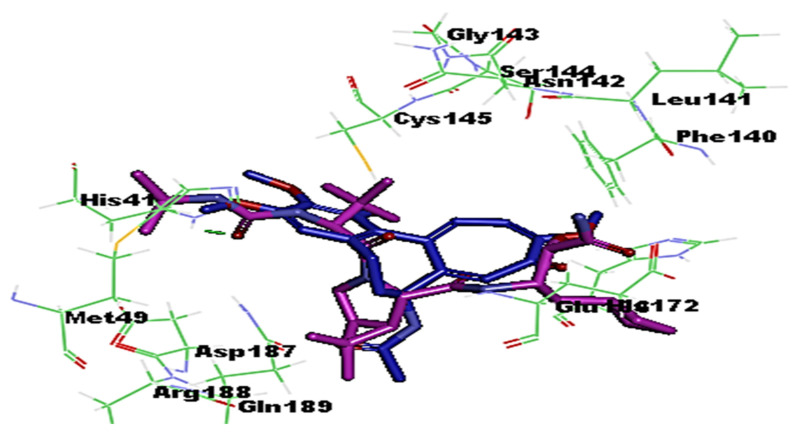
Alignment of Colchicine (blue) with Boceprevir (protease inhibitor) (purple) in the protease active site of SARS-CoV-2.

The coronaviral protease and RdRp are attractive antiviral drug targets because they are essential for coronaviral replication and genomic transcription. These two enzymes are considered promising drug targets in the discovery of specific anti–SARS-CoV-2 drugs [27]. Molecular docking is an important part of the drug design and discovery to identify interactions between the key amino acids of the target protein and the ligand. In the present study, Discovery studio 4.1 software [28] was used to perform molecular docking simulations to estimate the binding affinity of the Colchicine–receptor complex. The structure of the Protease enzyme complexed with Boceprevir (PDB ID: 6xqu) [29] and RdRp (PDB ID: 7b3c) [30] of SAR-SCoV-2 were retrieved from the Protein Data Bank (www.rcsb.org). The protein structures were minimized and prepared for the docking using a minimized and refined protein algorithm also; colchicine was constructed and prepared using a Ligand preparation protocol and energy minimized using the Merck Molecular Force Field (MMFF94) [28]. Molecular modeling simulation study was performed through docking of Colchicine in the binding site of the protease and RdRp of SARS-CoV-2 using C-Docker protocol, to gain insights into the binding mode and crucial molecular interactions of colchicine. The analysis of their binding modes was performed to predict the biological activity and to achieve further insight into binding orientations and interactions. Colchicine interacting with the protease and RdRp could potentially interfere with virus replication. Therefore, a molecular docking study was performed to identify and understand the interaction and binding affinity of colchicine with the protease and RdRp of SARS-CoV-2. The outcome of our docking study revealed that Colchicine gave a C-DOCKER interaction energy of 64.26 and 47.53 (Kcal/mol) with protease and RdRp, respectively. This finding indicated a higher binding stability for Colchicine–protease complexes than Colchicine–RdRp complex. In other words, Colchicine favored interactions with protease, involved seven hydrogen bonds, six hydrogen acceptors with Asn142, Gly143, Ser144, and Glu166, and one hydrogen-bond donors with Cys145. In addition, there were hydrophobic interactions with His172, Glu166, and Arg188 (Figure 2), while the interaction of Colchicine with RdRp involved three hydrogen bonds, two hydrogen bond acceptor with His133 and Lys47, and one hydrogen bond donor with Ser907. In addition, there were hydrophobic interactions with Lys47 and Thr141 (Figure 3), which is like the reported interaction of the Ivermectin drug with RdRp [31]. Moreover, an alignment study was performed for Colchicine which showed good alignment with approximately the same pharmacophoric elements of the reference compound (Boceprevir) (Figure 4). This indicated that Colchicine has a high probability of binding to the protease enzyme of SARS-CoV-2, preventing it from replication.

## 4. Colchicine Pharmacokinetics

The mean oral bioavailability of colchicine is about 45% and, interestingly, the peripheral leukocytes remain its main compartment to accumulate [32]. In comparison to its concentration in plasma, colchicine potential to selectively accumulate in neutrophiles is about 16 times higher. This could be attributed to a deficiency in the expression of an efflux protein known as P-glycoprotein (P-gp) among neutrophils [33]. Colchicine is metabolized in the liver and intestine by cytochrome P450 3A4 (CYP3A4) and is eliminated into the bile and urine via P-gp. Accordingly, it is contraindicated to prescribe colchicine among patients with hepatic or renal deterioration and especially if they are receiving CYP3A4 or P-gp inhibitors [32].

At prescribed doses, colchicine is well tolerated despite of its narrow therapeutic index. The most common side effects are gastrointestinal ones that can occur in more than 20% of patients in addition to other rarer side effects, such as neuromyopathy that likely occurs with chronic daily use of colchicine [16]. Lopes et al. had reported on the effectiveness of colchicine maximum daily dose when taken as 0.5 mg three times daily for 5 days and then the same dose twice daily for another 5 days on reducing the length of hospital stay and need for supplemental oxygen therapy by a median of 7 and 4 days, respectively [34].

### 4.1. Rationale for using Colchicine in Controlling ARDS and MOD

The pulmonary manifestations of SARS-CoV-2 are broad and mainly range from viral pneumonia to fatal ARDS (15% of cases) that is characterized by sudden respiratory failure due to over activation of the host immune response [35]. Hyper-inflammatory immune response the so-called “cytokine storm” will eventually correlate with COVID-19 severity, leading to exacerbation of respiratory failure and MOD due to extensive tissue damage. Studies have revealed that overproduction of pro-inflammatory cytokines (mainly IL-1, IL-6, TNF-α, and interferon) in addition to other anti-inflammatory cytokines (IL-4 and IL-10), immune cells infiltrate, and serum proteins as C-reactive protein (CRP), Lactate Dehydrogenase H, ferritin, and *D*-dimers are closely related to poor outcome [36,37]. Of note, the synthesis of IL-6 induces the production of CRP, which is considered to be one of the first biomarkers associated with cytokine storm among COVID-19 patients [38]. In theory, colchicine expresses a wide array of anti-inflammatory activity that mainly targets the cytokine storm at different levels and additionally, as a significantly cheaper drug compared to the currently used antiviral agents, such as remdesivir or molnupiravir, with a great availability, it has lead us to explore its effect in improving and controlling ARDS and other extra pulmonary complications of COVID-19 [38].

In light of its anti-inflammatory activity, randomized clinical trials as the GRECCO-19 (NCT04326790) and the COLCORONA multicenter study (NCT04322682) had evaluated the effectiveness of colchicine in reducing inflammatory markers and thereby improving clinical outcomes [38]. The results were promising as a former study had shown a statistically significant improvement in clinical status [39], while the latter study had shown a lowered rate of mortality and hospitalization [40]. Indeed, another case control study had shown a statistically significant reduction of ferritin, CRP, and *D*-dimer with a p value of 0.012, 0.014, and 0.037, respectively [37]. So far, there are approximately 26 ongoing clinical studies on the effect of colchicine on improving the clinical status of COVID-19 patients [41]. Recently, a meta-analysis study that was conducted using several databases published from December 2019 until 26 August 2021 had reported on the efficacy of colchicine in reducing inflammatory biomarkers among moderate-to-severe patient cases of COVID-19. Of interest, they recommended conducting further clinical trials to ensure the effectiveness of colchicine as an adjuvant treatment for patients with COVID-19 [42].

On the other hand, a randomized control study conducted on fifty-six hospitalized patients, allocated to colchicine and sixty patients receiving placebo had reported on the ineffectiveness of colchicine in the treatment of severe COVID-19 despite being a safe drug [43]. Additionally, a meta-analysis of randomized controlled trials conducted by Mehta et al. proved a moderate quality evidence suggesting no benefit of addition of colchicine to the standard care regimen in patients with COVID-19 [44]. Another study, conducted between 27 November 2020 and 4 March 2021 showed that colchicine was not associated with reductions in mortality, duration of hospital stay, or risk of clinical complications [45]. Another study concluded that colchicine treatment neither improved the clinical status, nor the inflammatory response, over the standard treatment and advised that a preventive effect of colchicine on further clinical deterioration should be considered [46].

Accordingly, and based on these findings, much more well-designed multicenter studies are still required to direct clinicians upon the optimum use and duration of colchicine within hospitalized or non-hospitalized patients with COVID-19. In the context of the above rationale (immunomodulatory action and promising in silico antiviral activity), we hope to design the following multicenter clinical trial to evaluate the effectiveness of using colchicine as a prompt treatment for COVID-19. The inclusion criteria and patient settings are: Hospitalized COVID-19 patients (age ranging from 17–70) with a rising titer in biomarker profile namely IL1, IL-6, and TNFα. Rising titer could be determined by at least two discrete time-point measurement in any of the inflammatory markers.

Intervention: This study will depend on random distribution of participants into 3 groups (at 1:1:1 ratio).

Group 1: patient receiving maximum safety dose of colchicine alone.
Group 2: patient receiving maximum safety dose of colchicine in addition to standard treatment (combination therapy).
Group 3: patient receiving standard treatment alone (control group).

Primary outcomes:

Among the primary outcomes, one is to estimate the risk of clinical deterioration mainly through the need of mechanical ventilator or presence of cardiac arrhythmias. A second one is to estimate the length of hospital stay and to evaluate the risk of 30 day mortality rate. The third outcome is to report side effects associated with colchicine at the prescribed dose.

### 4.2. Potential Role of Colchicine in Suppressing Various Inflammatory Signaling Pathways and Their Associations with COVID-19

Mitogen-activated protein kinases (MAPK) are formerly known as extracellular signal-regulated kinases (ERK) and are sets of proteins located on the cell surface that mediate signal activation up on their binding to signal proteins to the nucleic acid of the cell [42]. The resulted signals activate the level of cellular protein expression producing certain cellular changes such as cell multiplication, proliferation, as well as inflammation, particularly during the COVID-19 infection [47,48,49]. Various studies have been reported on the potential role of colchicine for suppressing the MAPK signaling pathways and therefore, as a promising therapy to control the inflammation and clinically relevant complications associated with the activation of this pathway [50,51,52].

It was recently reported that neopterin (NPT), a protein synthesized by macrophages up on their activation by interferon gamma (INF-γ) has a potential role in mediating inflammation and various clinical complications associated with COVID-19 [53]. On the other hand, it was also reported that NPT has anti-inflammatory and antioxidant effects by down-regulating the expression of nuclear factor kappa B (NF-κB) signaling and NLRP3 inflammasomes and therefore overcome hyper-inflammation, oxidative stress, and accompanying organ failure [53]. Another study conducted by Itano et al. showed that colchicine was able to significantly inhibit the angiotensin II-induced fibroblast migration in vitro and attenuated the renal fibrosis in a murine unilateral ureteral obstruction model [54]. It was also reported that colchicine in combination with nicorandil was able to prevent monocrotaline-induced rat pulmonary arterial hypertension [55].

Signal transducers and activators of transcription (STATs) are a group of proteins that are important for cancer survival, proliferation, metastases, and angiogenesis [56]. A study conducted in 2020 by Tantawy et al. confirmed the IL6/JAK2/STAT3 axis down-regulation as a major contributor for inhibiting the progression of lung carcinoma via influencing the cell apoptosis [57]. Various reports have documented the potential role of colchicine in the suppression of this inflammatory cascade pathway and therefore predicting its potential use with lung cancer and COVID-19 cytokines storm [57,58]. Autophagy is a natural, mechanism of the cell to eliminate unnecessary or dysfunctional cellular components via a lysosome-dependent regulated manner [59]. Colchicine was recently reported to interfere with the autophagy mechanism and therefore enhance the efficiency of cell transfection by DNA, conferring its potential activities against cancer, the virally infected cells, and other clinically relevant pathological conditions [60,61,62].

Furthermore, many recent studies have reported the use of colchicine for protecting against acute myocardial infarction, cardiomyopathy [63], and other clinically relevant conditions in humans such as renal ischemia, liver damage, and osteoarthritis [64,65] by modulating the macrophage polarization and suppressing the TLR4/NFκB/NLRP3 signal pathway and therefore, inhibiting pyroptosis and inflammatory response [63,64,65]. Recently, it was also reported that the cluster of differentiation 147 (CD147) transmembrane protein is a novel route for SARS-CoV-2 entry and therefore influences viral pathogenesis in humans [66]. A recent study conducted by Avolio et al. revealed that the SARS-CoV-2 Spike protein disrupts human cardiac pericytes’ function through CD147-receptor-mediated signaling [67]. However, the potential role of colchicine in suppressing such mechanism has not yet been confirmed.

### 4.3. Colchicine Dosage, Timing, and Drug–Drug Interaction in COVID-19 Positive Patients

Colchicine is a well-tolerated drug and the most common side effects are gastrointestinal, including diarrhea and colitis that can occur in more than 20% of patients in addition to other rarer side effects such as neuromyopathy that likely occurs with chronic daily use of colchicine [16]. It was previously reported that the colchicine maximum daily dose when taken as 0.5 mg three times daily for 5 days and then same dose twice daily for another 5 days reduces clinical complications by a median of 7 and 4 days, respectively [34]. Recently, Vitiello et al. reported that Low-dose colchicine could be considered safe and effective for controlling of the cytokine storm in patients with COVID-19, especially as an adjunctive medication to other treatment options [68].

A meta-analysis study on colchicine’s safety profile during the pre-COVID-19 time revealed that colchicine is a well-tolerated drug and has a good safety profile, except for the occurrence of gastrointestinal disorder [69]. Similar findings were observed in other meta-analysis pooled models among patients with COVID-19 [44,70]. It was reported that the metabolism of Colchicine can be decreased when combined with Remdesivir [71,72]. It was also reported that a fatal interaction occurred upon the coadministration of colchicine with p-glycoprotein and cytochrome P450 inhibitors such as ketoconazole, lopinavir, nelfinavir, ritonavir cyclosporine, and macrolides such as erythromycin and clarithromycin [73].

## 5. Conclusions

The clinical progression of COVID-19 towards a more severe scenario is linked to the production of a cytokine storm. Colchicine has a great potential to inhibit the cytokine storm through interfering with several pro/anti-inflammatory pathways and NLRP3 activation. Docking analysis provided strong evidence of antiviral activity comparable to that of a standard antiprotease drug, boceprevir, by inhibiting the protease enzyme of SARS-CoV-2, and therefore, could prevent its replication. Nevertheless, the use of colchicine in the treatment of COVID-19 patients remains questionable. Hence, comprehensive clinical trials are required to show the effectiveness of repurposing colchicine, the old and low-cost anti-inflammatory drug, as a prompt treatment for patients with COVID-19.
